# Characteristics of Neuromuscular Control of the Scapula after Stroke: A First Exploration

**DOI:** 10.3389/fnhum.2014.00933

**Published:** 2014-11-17

**Authors:** Liesbet De Baets, Ellen Jaspers, Luc Janssens, Sara Van Deun

**Affiliations:** ^1^REVAL Rehabilitation Research Center – BIOMED Biomedical Research Institute, Faculty of Medicine and Life Sciences, Hasselt University, Diepenbeek, Belgium; ^2^Neural Control of Movement Laboratory, ETH Zurich, Zurich, Switzerland; ^3^Faculty of Industrial Engineering Sciences, KU Leuven, Leuven, Belgium

**Keywords:** scapula, muscle timing, stroke, recruitment patterns, rehabilitation

## Abstract

This study aimed to characterize scapular muscle timing in stroke patients with and without shoulder pain. Muscle activity of upper trapezius, lower trapezius, serratus anterior, infraspinatus, and anterior deltoid (AD) was measured (Delsys Trigno surface EMG system, USA) in 14 healthy controls (dominant side) and 30 stroke patients (hemiplegic side) of whom 10 had impingement-like shoulder pain. Participants performed 45° and full range anteflexion, in two load conditions. The impact of group, anteflexion height, load condition, and muscle was assessed for onset and offset of the different muscles relative to the onset and offset of AD, using a 3 (group) × 2 (height) × 2 (load) × 4 (muscle) mixed model design. Recruitment patterns were additionally described. Across all load conditions and groups, serratus anterior had a significantly earlier onset and, together with lower trapezius, a significantly later offset in 45° compared to full range anteflexion tasks (*p* < 0.001). In stroke patients without pain, lower trapezius had furthermore a significantly earlier onset in comparison to stroke patients with shoulder pain (all tasks, *p* = 0.04). Serratus anterior also showed a significantly earlier offset in stroke patients with shoulder pain in comparison to controls (*p* = 0.01) and stroke patients without pain (*p* < 0.001). Analysis of muscle recruitment patterns indicated that for full range tasks, stroke patients without pain used early and prolonged activity of infraspinatus. In stroke patients with shoulder pain, recruitment patterns were characterized by delayed activation and early inactivity of serratus anterior. These timing results can serve as a reference frame for scapular muscle timing post-stroke, and when designing upper limb treatment protocols and clinical guidelines for shoulder pain after stroke.

## Introduction

Shoulder pain is a common complication after stroke, affecting one-third of stroke patients in general. It not only negatively impacts on a patient’s independency level and functional ability but also impedes a successful rehabilitation (Roy et al., 1994).

Apart from decreased glenohumeral motion, spasticity, subluxation, and somatosensory impairments (Lindgren et al., 2012, 2014; Lindgren and Brogårdh, 2014), poor scapulothoracic position, and aberrant scapulohumeral motion are also considered risk factors in the development of shoulder dysfunction and pain after stroke (Niessen et al., 2008; Hardwick and Lang, 2011). The ability to adapt the scapulothoracic position to the degree of humerothoracic movement during arm anteflexion relies upon adequate timing of specific scapular musculature. A stable scapulohumeral joint requires the synchronized control of the scapulothoracic and glenohumeral stabilizing muscles relative to prime mover muscle activity (Wickham et al., 2010; Phadke and Ludewig, 2013). The association between altered scapular muscle activity, and the occurrence of impingement pain has been extensively studied in non-stroke subjects during arm elevation (Ludewig and Cook, 2000; Moraes et al., 2008; Ludewig and Braman, 2011; De Baets et al., 2013; Larsen et al., 2013; Phadke and Ludewig, 2013; Worsley et al., 2013; Struyf et al., 2014). However, results of these studies are not conclusive. For example, Moraes et al. (2008) found a delayed onset of lower trapezius in patients with impingement, whereas Worsley et al. (2013) additionally reported a delayed onset of serratus anterior, and an earlier offset of serratus anterior and lower trapezius, and Padke and Ludewig (2013) furthermore reported an earlier activation of upper trapezius. In contrast, Larsen et al. (2013) did not find any changes in onset time of these muscles in persons with impingement. Moreover, apart from the discrepancies regarding muscle timing, results on shoulder muscle recruitment patterns are also highly variable. While several authors have reported a comparable pattern of muscle activation (upper trapezius activity, followed by serratus anterior and lower trapezius) in persons with and without shoulder pathology (Wadsworth and Bullock-Saxton, 1997; Moraes et al., 2008; Struyf et al., 2014), others did find alterations in recruitment patterns (Phadke and Ludewig, 2013). More specifically, in pain free persons, the latter authors (Phadke and Ludewig, 2013) reported activity of anterior deltoid (AD), followed by serratus anterior, upper trapezius, and lower trapezius during unloaded arm anteflexion. In persons with impingement pain, upper trapezius activity was followed by AD, serratus anterior, and lower trapezius.

To our knowledge, no evidence currently exists on how residual motor impairments in stroke patients, who have developed the ability to perform isolated and selective arm movements, might create imbalances in scapulothoracic and scapulohumeral muscle coordination during upper limb tasks (De Baets et al., 2013). Such information could be of interest when studying the impact of the scapulothoracic and glenohumeral joints as contributing factors to upper limb (dys)functions (i.e., decreased range of motion) and pain after stroke. There is furthermore a lack of evidence regarding the influence of external load and anteflexion height on the activation patterns of scapular muscles post-stroke. An earlier activation and a delayed deactivation of lower trapezius and serratus anterior were already detected in loaded conditions during raising and lowering of the arm, respectively, in healthy persons and persons with impingement (Phadke and Ludewig, 2013).

Knowledge of typical temporal patterns of shoulder muscle activity in stroke patients with and without shoulder pain, and the influence of specific parameters, can be of value for physical therapists designing upper limb treatment programs. Therefore, the goal of this study is to characterize the typical temporal patterns of scapular muscle activity in stroke patients with and without shoulder pain as compared to healthy controls, by means of electromyography during low and high, unloaded and loaded anteflexion tasks. We hypothesize altered recruitment patterns and muscle timing of stabilizing musculature in stroke patients with shoulder pain as compared to healthy controls and patients without shoulder pain. Furthermore, an earlier activation, and later deactivation of scapulothoracic musculature in high versus low anteflexion tasks and in loaded versus unloaded tasks, is hypothesized.

## Materials and Methods

### Participants

Stroke patients were recruited from different rehabilitation centers in Flanders (Belgium) and were considered eligible for participation in case they (1) were at least 6 weeks after a first time stroke (cortical or subcortical lesion); (2) had mild to moderate upper limb motor impairment (score of ≥30 on the Fugl-Meyer upper limb motor part (Platz et al., 2005)); and (3) were able to perform 45° of active humerothoracic anteflexion (measured with goniometry). Stroke patients with shoulder pain were included when they (1) experienced anterolateral shoulder pain during daily activities with a painful arc during 60°–120° of arm anteflexion for at least four weeks since stroke onset; and (2) had a positive Neer impingement test, i.e., reported pain when the humeral greater tuberosity was impacted against the inferior acromion (Neer and Foster, 1980). Healthy controls without self-reported shoulder pain were recruited via family and relatives. For all participants, following exclusion criteria were applied: (1) body mass index higher than 28; (2) inability to understand the instructions; (3) known history of shoulder and/or neck pain or discomfort in the last six months prior to stroke; (4) an event of shoulder dislocation, fracture or surgery during life time; or (5) other systemic and/or neurologic diseases. All stroke patients received standard care and physiotherapy, attuned to their specific needs. An overview of participants’ characteristics is given in Table 1.

**Table 1:** **Participants’ characteristics**.

	Healthy controls	Stroke patients without shoulder pain	Stroke patients with shoulder pain
Subjects			
number	14	20	10
Male/female	10/4	14/6	9/1
Age (years) (mean ± SD)	61 ± 11	59 ± 11	62 ± 21
Body mass index (mean ± SD)	24 ± 2	25 ± 2	25 ± 2
Dominant side (left/right)	2/12	1/19	1/9
Hemiplegic side (left/right)	–	9/11	5/5
Time since stroke (weeks) (mean ± SD)	–	24 ± 18	22 ± 7
Lesion location (cortical/subcortical)	–	15/5	8/2
Fugl-Meyer score^a^ (mean ± SD)	–	53 ± 7	51 ± 7

*^a^Upper limb motor section, maximal 66*.

All participants gave informed consent, as approved by the Ethical Committee of the University Hospital Leuven (Belgium), prior to study participation.

### Measurement procedure

Scapular muscle activity was recorded using surface electromyography (Delsys Trigno EMG system, Boston, MA, USA) at the hemiplegic side. Following muscles were measured: upper trapezius (midpoint of the line between angulus acromialis and C7 processus spinosus), lower trapezius (at one-third of the line between trigonum scapula and T8 processus spinosus), serratus anterior (anterior to the latissimus dorsi at the level of the scapular inferior angle), infraspinatus (approximately 4 cm below the spine, over the infrascapular fossa, parallel to the scapular spine), and AD (2–4 cm below the lateral clavicula, parallel to the muscle fibers) (Cram and Kasman, 1998; Hermens et al., 2009). To ensure consistency of EMG-sensor position, these were placed by the same investigator for all participants. Prior to sensor placement, the skin over the muscle of interest was prepared and cleaned with alcohol. Muscle activity was recorded with a sampling rate of 2000 Hz. Correct sensor positioning and signal quality was verified by visual inspection of the EMG-signals during muscle specific movements.

The movement protocol consisted of anteflexion tasks from 0° to 45° and from 0° to maximal anteflexion, all executed while seated. These anteflexion tasks were performed under an unloaded condition and a loaded condition. Every task consisted of 12 consecutive repetitions. For the loaded conditions, a dumbbell weighting 1–1.25 kg (calculated relative to body weight) was attached to the participants’ wrist (average weight 1.15 kg). A bar was placed in front of the patient at 45° of anteflexion to give visual information about the correct anteflexion height for this task. Participants were instructed to start with the elbow fully extended and thumb pointing upward, and to maintain this positioning during the anteflexion. To ensure correct task performance and a proper pace of task execution (1 s up, 1 s down, 3 s rest for 45° tasks; 3 s up, 3 s down, 4 s rest for full range tasks), each participant was given some practice trials.

### Data-analysis

From the recorded trials, only the middle 10 repetitions were selected for data-analysis, as these were considered free from initiation or completion strategies. Raw EMG data were first high-pass filtered with a sixth-order Butterworth filter of 20 Hz to avoid movement and cardiac artifacts, and subsequently rectified and filtered with a low-pass filter (cut-off frequency of 45 Hz) to smooth the data. Both filters were implemented as bidirectional filters to reduce the phase error.

**Figure 1:**
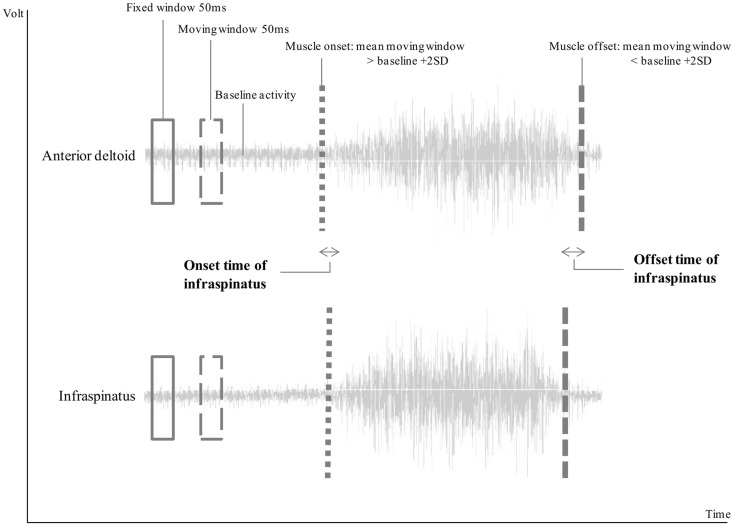
**Visual representation of the calculation of muscle onset and offset**. In this example, infraspinatus was active after the onset of anterior deltoid, resulting in negative onset time, and inactive before the offset of the anterior deltoid, resulting in a positive offset time.

Onset of muscle activity was defined as an increase in muscle activity of more than two SD on top of the mean baseline activity (as recorded for 10 s prior to movement start) for at least 50 ms. Muscular offset was reached when the recorded activity was lower than the mean baseline activity plus two SD for at least 50 ms (Hodges and Bui, 1996). Each calculated onset and offset was visually checked for erroneous signals due to cardiac or other motion artifacts (Di Fabio, 1987). Parameters of interest were (1) time of muscular onset, and (2) time of muscular offset of stabilizing musculature (upper trapezius, lower trapezius, serratus anterior, and infraspinatus) relative to time of the onset and offset, respectively, of the prime mover for anteflexion (AD) (Figure 1). Data of these two timing parameters were compared between the different groups, height and load conditions, and muscles. A positive latency time (milliseconds) indicated activity prior to AD onset and inactivity prior to AD offset.

Lastly, the sequence in time of onset and time of offset of the different muscles was analyzed per group (based on average group data). Afterwards, sequences of recruitment were further explored for each task at the level of the individual subject within each group. Given that the sampling rate of the EMG-signal was set at 2000 Hz (which implies a time resolution of 0.5 ms), time intervals could theoretically be measured with an accuracy of 1 ms. Even though bidirectional filters were used to reduce timing errors, an uncertainty error of 4 ms was taken into account. Therefore, the accuracy for determining the onset time was estimated at 10 ms, meaning that the difference in timing between two muscles within one individual had to exceed 10 ms to represent real difference in recruitment timing.

### Statistical analysis

Differences in onset and offset timing between groups (stroke patients without shoulder pain, stroke patients with shoulder pain and controls), anteflexion heights (45°, full range), load conditions (unloaded, loaded), and muscles (infraspinatus, upper trapezius, lower trapezius, and serratus anterior) were analyzed using a 3 (Group) × 2 (height) × 2 (load) × 4 (muscle) Mixed Model (Littell et al., 2002). As such, four main effects and six interaction effects were calculated for onset timing and offset timing separately. The use of mixed model analysis was preferred as this analysis is robust in analyzing semi-normally distributed data (Verbeke and Lesaffre, 1997), allows for repeated measures analysis, and can handle missing data (which was the case for on/offset data of some participants). All statistics were done in SAS software version 9.4 Foundation and enterprise guide.

## Results

### Timing parameters of stabilizing muscles relative to anterior deltoid timing

In three stroke patients with shoulder pain, no onset/offset could be detected for serratus anterior and lower trapezius in 45° anteflexion tasks and data for these muscles in this task was limited to seven patients only.

A significant main effect for height (*p* < 0.001) and muscle (*p* < 0.0001), and a significant interaction effect for height × muscle (*p* = 0.02) were observed for onset and offset.

*Post hoc* analysis of the *height* × *muscle* interaction effect for onset indicated that, for all load conditions and groups, all muscles had a significantly different onset (all *p* < 0.005). Only serratus anterior and lower trapezius (both heights), and serratus anterior and infraspinatus (45° task) did not differ significantly from each other. *Post hoc* analysis further indicated that only serratus anterior had a significant earlier onset in 45° compared to full range tasks (*p* < 0.0001).

*Post hoc* analysis of the height × muscle interaction effect for offset indicated that, over all load conditions and groups, all muscles had a significantly different offset (*p* < 0.005) except for infraspinatus and upper trapezius (both heights). This analysis also showed that only serratus anterior and lower trapezius had a significant earlier offset in full range compared to 45° anteflexion tasks (*p* < 0.001).

A significant *group* × *muscle* interaction effect was also found for onset and offset. *Post hoc* analysis for onset firstly indicated that, over all tasks, lower trapezius had a significant earlier onset in stroke patients without pain than in stroke patients with shoulder pain (*p* = 0.04). Second, for each group, all muscles showed a significantly different onset (*p* < 0.001), except for (1) infraspinatus and serratus anterior in controls, (2) infraspinatus and lower trapezius, and serratus anterior and lower trapezius in stroke patients without shoulder pain, and (3) serratus anterior and lower trapezius in stroke patients with shoulder pain.

*Post hoc* analysis for offset indicated that, over all tasks, serratus anterior had a significant earlier offset in stroke patients with shoulder pain compared to controls (*p* = 0.01) and stroke patients without shoulder pain (*p* < 0.001). Furthermore, all muscles showed a significantly different offset for every group (*p* < 0.001), except for (1) infraspinatus and serratus anterior, and infraspinatus and upper trapezius in controls, (2) infraspinatus and upper trapezius in stroke patients without shoulder pain, and (3) serratus anterior and lower trapezius, and infraspinatus and upper trapezius in stroke patients with shoulder pain.

### Recruitment patterns

#### Recruitment patterns based on average group data

In controls and stroke patients with shoulder pain, upper trapezius was activated first during each task, while lower trapezius was activated last. In stroke patients without shoulder pain, upper trapezius was also active first, but lower trapezius was activated last only during the 45°, unloaded task. In all other tasks, serratus anterior was activated last in this group. The difference in onset between upper trapezius and lower trapezius, and upper trapezius and serratus anterior was significant across all groups and tasks (all *p* < 0.001).

In every group, lower trapezius was also the first muscle that was inactive and upper trapezius the last. Only in stroke patients with shoulder pain, during the 45° tasks, serratus anterior was inactive first (in the loaded condition together with lower trapezius), while in the full range loaded task infraspinatus was inactive last. The difference in offset between upper trapezius and lower trapezius, upper trapezius and serratus anterior, and lower trapezius and infraspinatus was significant across all groups and tasks (all *p* < 0.0001). Recruitment patterns based on average group data are visualized for every task in Supplementary Material (Presentation S1 in Supplementary Material).

#### Muscle recruitment patterns based on individual data

Owing to large SD of group onset/offset data (Table 2), recruitment patterns were further explored based on individual muscle activation patterns per task. This was accomplished by categorizing each muscle’s onset/offset timing as activity/inactivity before, after or together with the onset/offset of AD. Results of this individual data exploration are found in Table 3. As group differences in average onset and offset recruitment patterns were mainly found in the sequence of infraspinatus, serratus anterior and AD, we will focus on these muscles only.

**Table 2:** **Mean with standard deviation (SD) of timing parameters for the all anteflexion tasks, expressed in milliseconds**.

		45°Anteflexion, unloaded						45° Anteflexion, loaded					
		Controls		Stroke no pain		Stroke pain		Controls		Stroke no pain		Stroke pain	
		ON	OFF	ON	OFF	ON	OFF	ON	OFF	ON	OFF	ON	OFF
Upper trapezius	Mean (SD)	115 (219)	−70 (341)	75 (168)	−371 (557)	152 (218)	−151 (415)	79 (133)	−97 (271)	35 (128)	−162 (502)	178 (229)	−235 (474)
Infraspinatus	Mean (SD)	−63 (143)	80 (351)	8 (89)	−231 (402)	−8 (241)	−145 (444)	−80 (121)	26 (260)	−36 (143)	−128 (368)	10 (201)	−99 (503)
Lower trapezius	Mean (SD)	−236 (201)	419 (318)	−35 (152)	290 (440)	−135 (134)	338 (388)	−172 (169)	305 (280)	−92 (175)	419 (326)	−246 (254)	436 (551)
Serratus anterior	Mean (SD)	48 (144)	114 (437)	−7 (162)	−307 (495)	−5 (255)	380 (639)	29 (133)	15 (382)	−143 (179)	−108 (550)	−119 (249)	429 (206)

		**Full range anteflexion, unloaded**						**Full range anteflexion, loaded**					
		**Controls**		**Stroke no pain**		**Stroke pain**		**Controls**		**Stroke no pain**		**Stroke pain**	
		**ON**	**OFF**	**ON**	**OFF**	**ON**	**OFF**	**ON**	**OFF**	**ON**	**OFF**	**ON**	**OFF**

Upper trapezius	Mean (SD)	254 (336)	−151 (733)	115 (252)	−351 (257)	114 (245)	−74 (163)	56 (370)	−49 (654)	−24 (146)	−224 (647)	122 (280)	−364 (491)
Infraspinatus	Mean (SD)	−38 (297)	−29 (546)	5 (204)	−276 (460)	−19 (205)	20 (284)	−69 (290)	−30 (460)	−151 (499)	−261 (439)	−34 (205)	−12 (560)
Lower trapezius	Mean (SD)	−265 (281)	789 (794)	−140 (173)	843 (614)	−398 (704)	889 (884)	−206 (296)	666 (617)	−96 (142)	639 (682)	−249 (500)	985 (801)
Serratus anterior	Mean (SD)	164 (553)	421 (906)	−321 (335)	240 (717)	−279 (836)	836 (502)	−84 (385)	237 (680)	−184 (253)	236 (743)	−352 (432)	766 (748)

**Table 3:** **Group-specific classifications of onset and offset time relative to anterior deltoid (AD) onset and offset time per task**.

		45°Anteflexion unloaded			45°Anteflexion loaded			Full anteflexion unloaded			Full anteflexion loaded		
		Controls	Stroke no pain	Stroke pain	Controls	Stroke no pain	Stroke pain	Controls	Stroke no pain	Stroke pain	Controls	Stroke no pain	Stroke pain
**ONSET**													
Upper trapezius	Before AD	75%	67%	80%	67%	56%	80%	71%	71%	30%	43%	61%	60%
	After AD	25%	33%	20%	33%	44%	20%	29%	29%	70%	50%	39%	40%
	Together with AD	–	–	–	–	–	–	–	–	–	7%	–	–
Lower trapezius	Before AD	10%	43%	10%	8%	31%	50%	14%	22%	30%	21%	22%	30%
	After AD	90%	57%	60%	92%	69%	20%	86%	72%	70%	79%	78%	70%
	Together with AD	–	–	–	–	–	–	–	6%	–	–	–	–
	No onset	–	–	30%	–	–	30%	–	–	–	–	–	–
Serratus anterior	Before AD	67%	67%	38%	73%	25%	22%	39%	17%	10%	39%	22%	10%
	After AD	33%	33%	25%	27%	69%	45%	61%	83%	90%	61%	67%	90%
	Together with AD	–	–	–	–	6%	–	–	–	–	–	11%	–
	No onset	–	–	37%	–	–	33%	–	–	–	–	–	–
Infraspinatus	Before AD	45%	53%	50%	31%	35%	40%	21%	60%	20%	14%	37%	20%
	After AD	55%	35%	20%	69%	55%	40%	64%	30%	50%	79%	58%	70%
	Together with AD	–	12%	30%	–	10%	20%	15%	10%	30%	7%	5%	10%
**OFFSET**													
Upper trapezius	Before AD	42%	26%	40%	42%	31%	30%	43%	6%	30%	50%	37%	30%
	After AD	58%	74%	60%	58%	69%	70%	57%	94%	70%	50%	63%	70%
	Together with AD	–	–	–	–	–	–	–	–	–	–	–	–
Lower trapezius	Before AD	90%	71%	40%	75%	89%	50%	93%	94%	90%	86%	84%	100%
	After AD	10%	21%	30%	25%	11%	20%	7%	6%	10%	14%	16%	–
	Together with AD	–	8%	–	–	–	–	–	–	–	–	–	–
	No onset	–	–	30%	–	–	30%	–	–	–	–	–	–
Serratus anterior	Before AD	55%	28%	50%	55%	35%	67%	77%	69%	100%	69%	58%	90%
	After AD	45%	72%	13%	45%	65%	–	23%	31%	–	31%	42%	10%
	Together with AD	–	–	–	–	–	–	–	–	–	–	–	–
	No onset	–	–	37%	–	–	33%	–	–	–	–	–	–
Infraspinatus	Before AD	66%	32%	50%	54%	45%	50%	43%	26%	70%	43%	39%	70%
	After AD	34%	68%	50%	46%	55%	40%	50%	74%	20%	50%	61%	30%
	Together with AD	–	–	–	–	–	10%	7%	–	10%	7%	–	–

Concerning *onset timing* for the 45° anteflexion tasks, serratus anterior activity prior to or together with AD was seen in 67% (unloaded) and 73% (loaded) of controls, in 67% (unloaded) and 31% (loaded) of stroke patients without shoulder pain, and in 38% (unloaded) and 22% (loaded) of patients with shoulder pain. Infraspinatus was active before or together with AD in 45% (unloaded) and 31% (loaded) of controls, in 65% (unloaded) and 45% (loaded) of stroke patients without shoulder pain, and in 80% (unloaded) and 60% (loaded) of stroke patients with shoulder pain.

For full range anteflexion tasks, serratus anterior activated before or together with the AD in 39% (unloaded and loaded) of controls, in 17% (unloaded) and 33% (loaded) of stroke patients without shoulder pain, and in 10% (unloaded and loaded) of patients with shoulder pain. Infraspinatus was active prior to or together with AD in 36% (unloaded) and 21% (loaded) of controls, in 70% (unloaded) and 42% (loaded) of stroke patients without shoulder pain and in 50% (unloaded) and 30% (loaded) of stroke patients with shoulder pain.

For *offset timing* during the 45° anteflexion tasks, serratus anterior was inactive before or together with the offset of AD in 55% (unloaded and loaded) of controls, in 28% (unloaded) and 35% (loaded) of stroke patients without shoulder pain, and in 50% (unloaded) and 67% (loaded) of patients with shoulder pain. Infraspinatus was inactive before or together with AD in 66% (unloaded) and 54% (loaded) of controls, in 32% (unloaded) and 45% (loaded) of stroke patients without shoulder pain and in 50% (unloaded) and 60% (loaded) of stroke patients with shoulder pain.

In full range tasks, serratus anterior was inactive before or together with the offset of AD in 77% (unloaded) and 69% (loaded) of controls, in 69% (unloaded) and 58% (loaded) of stroke patients without shoulder pain, and in 100% (unloaded) and 90% (loaded) of patients with shoulder pain. Infraspinatus stopped before or together with AD in 50% (unloaded and loaded) of controls, in 26% (unloaded) and 39% (loaded) of stroke patients without shoulder pain and in 80% (unloaded) and 70% (loaded) of stroke patients with shoulder pain.

## Discussion

When moving the arm, our muscles exhibit a feed-forward or anticipatory control activity to ensure that the scapular position is adapted to the humeral position. As such, the scapula serves as a stable base for arm anteflexion. Motor control of the shoulder relies on a synchronized activation of the upper trapezius, lower trapezius, and serratus anterior (Inman et al., 1944; Ebaugh et al., 2005) to upwardly rotate the scapula. Next, this scapulothoracic force couple works in coordination with the humeral elevators (AD) and thereby offers an optimal tension-force relation for glenohumeral muscles such as infraspinatus. Infraspinatus activity counterbalances the upward force of the AD on the humerus, and thus, centers the humeral head in the glenoid fossa. As post-stroke hemiparesis might provide inadequate conditions for selective muscle activation, this study wanted to assess alterations in this feed-forward control in stroke patients and investigate their relation to the development of shoulder pain by means of muscular timing assessments (EMG). Additionally, the impact of anteflexion height and load was assessed. Tasks were chosen based on the clinical evaluation of the shoulder and their relevance in daily life.

Across all tasks, we found a delayed onset for lower trapezius in stroke patients with shoulder pain as compared to stroke patients without shoulder pain. Inspection of the individual recruitment patterns further indicated that, especially in the low anteflexion tasks, a high percentage of stroke patients with pain had no or a delayed serratus anterior onset. This was not seen in controls or stroke patients without pain. Serratus anterior was moreover earlier inactive in stroke patients with shoulder pain compared to controls and stroke patients without shoulder pain, across all tasks. This was also confirmed in the inspection of individual recruitment patterns, i.e., more stroke patients with shoulder pain showed inactivity of serratus anterior before the offset of AD, compared to the other two groups. The alterations in stroke patients with shoulder pain are quite similar to the delayed serratus anterior and lower trapezius onset (Moraes et al., 2008; Worsley et al., 2013), and earlier serratus anterior offset (Worsley et al., 2013) seen in persons with impingement. The pull of serratus anterior and lower trapezius ensures a stable scapulothoracic joint and seems necessary to reduce the risk for subacromial impingement, probably by providing adequate scapular lateral rotation and posterior tilting (Ludewig and Cook, 2000). This pull might furthermore allow for proper muscle activation of the rotator cuff. It appears that stroke patients without shoulder pain use a strategy of early infraspinatus activity, in comparison to stroke patients with shoulder pain and healthy controls. They also showed a longer activity of the infraspinatus relative to AD’s offset, as compared to stroke patients with shoulder pain. These results point toward the role of the timing of the infraspinatus to adequately stabilize the shoulder and prevent shoulder pain in stroke patients. The stereotyped pattern of early and prolonged infraspinatus activation is thus likely to guarantee sufficient subacromial space, probably by pulling the humerus downward during anteflexion, and this knowledge should be taken into account during rehabilitation to prevent impingement.

In contrast to our hypothesis and other studies who reported alterations in timing of lower trapezius and serratus anterior during loaded arm anteflexion in healthy persons and persons with impingement (Phadke and Ludewig, 2013), our study could not identify significant effects of the loading. However, we did show effects of anteflexion height. Across all groups, serratus anterior was earlier active, and, together with lower trapezius, later inactive in low versus high tasks. These results additionally stress the importance of the serratus anterior and lower trapezius, especially during low anteflexion tasks, to keep the scapula still on the thorax, i.e., to ensure scapular setting. Since subacromial impingement is already possible at low anteflexion angles (Bey et al., 2007), this setting is essential to avoid compression of soft tissue structures in the subacromial space. The early activation of serratus anterior activates and its prolonged activity in low versus high anteflexion tasks might counteract the passive scapular movement caused by posterior glenohumeral structures (e.g., joint capsular structures, teres major, lattisimus dorsi, infraspinatus) when the humerus moves, and thereby keep the scapula still on the thorax.

The lack of uniform data on muscle recruitment patterns in healthy controls and persons with impingement in previous literature (De Baets et al., 2013; Struyf et al., 2014) and the high inter-subject variability we found within each study group clearly show that it is not recommended to describe typical recruitment patterns based on average data. We therefore considered the individual-specific patterns of recruitment to further interpret our data and compare this to previous literature. Such approach has been used recently to describe scapulothoracic kinematics in healthy controls (Lawrence et al., 2014).

A limitation of the current study is that we only included stroke patients who had developed the ability to move outside the synergetic pattern and to perform analytical anteflexion movements. As a consequence, results should not be generalized to patients who move in the abnormal, stereotypical pattern of elbow flexion with shoulder abduction–extension-external rotation, or elbow extension with adduction–flexion-internal rotation (Twitchell, 1951). Furthermore, we did not account for the side and type of stroke or the amount of brain damage. Lastly, the use of surface EMG is prone to signal noise by motion or cardiac artifacts, which resulted in some data loss, i.e., 15% lost trials for upper trapezius, 16% for lower trapezius, 21% for serratus anterior, and 7% for infraspinatus. Combining EMG data with scapular kinematics is indispensable to further increase our knowledge of upper limb recovery post-stroke, and to clarify why some patients do and others do not develop compensations and/or shoulder pain.

Current study results paved the road to gain a deeper understanding on alterations in recruitment and the emergence of compensatory strategies in the scapular and glenohumeral stabilizers post-stroke. We identified the presence of compensatory motor control of infraspinatus during arm anteflexion in stroke patients without shoulder pain. Our results furthermore indicated that stroke patients with shoulder pain should relearn scapular motor control, mainly of the serratus anterior and lower trapezius to address the scapular setting and to restore dynamic stability while doing arm movements. Future treatment guidelines for scapular motor control and shoulder muscle strengthening should moreover consider the impact of a task demand, i.e., reaching height. As important stabilizers are already active in low anteflexion tasks to perform a scapular setting, this task is deemed highly suited for the training of temporal muscle timing early after stroke when patients may still have mobility problems in higher degrees. Motivating patients to perform arm anteflexion tasks in higher degrees in occupational or physical therapy should be done cautiously and only after a good control of scapular setting and rotation in lower anteflexion tasks has been established.

This knowledge has the potential to offer a useful way for clinicians to prescribe appropriate therapeutic management strategies and for researchers to enhance knowledge in relation to this clinical challenge.

## Author Contributions

All authors met following criteria: substantial contributions to the conception or design of the work; or the acquisition, analysis, or interpretation of data for the work; drafting the work or revising it critically for important intellectual content; final approval of the version to be published; agreement to be accountable for all aspects of the work in ensuring that questions related to the accuracy or integrity of any part of the work are appropriately investigated and resolved.

## Conflict of Interest Statement

The authors declare that the research was conducted in the absence of any commercial or financial relationships that could be construed as a potential conflict of interest.

## Supplementary Material

The Supplementary Material for this article can be found online at http://www.frontiersin.org/Journal/10.3389/fnhum.2014.00933/abstract
