# Supplementary material for: Characteristics of Neuromuscular Control of the Scapula after Stroke: A First Exploration
Source: Front Hum Neurosci. 2014 Nov 17;8:933. doi: 10.3389/fnhum.2014.00933 (PMC4235078; doi:10.3389/fnhum.2014.00933)
Supplement: Supplementary file 1 [file Presentation1.PDF]

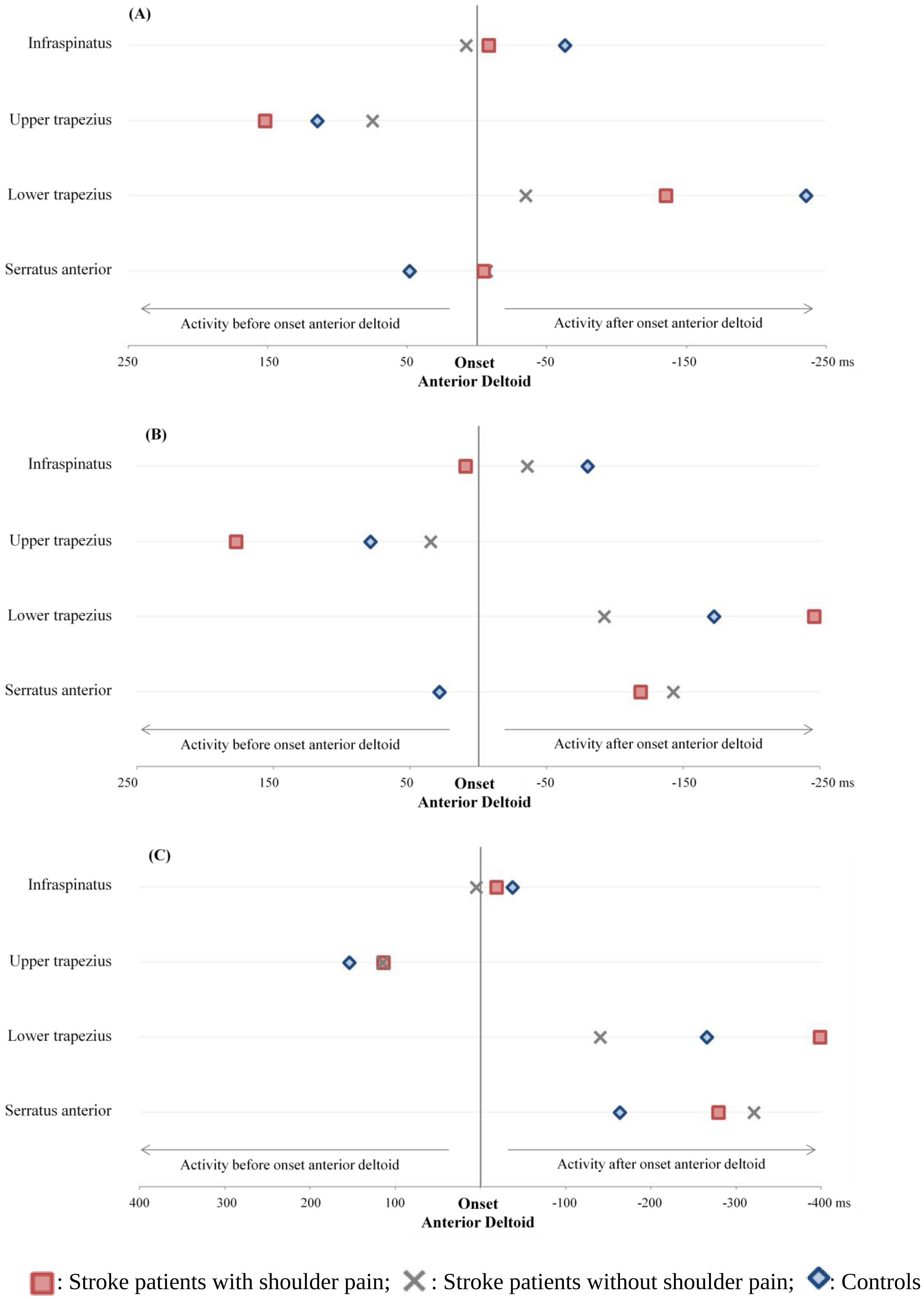
(A)
Infraspinatus
Upper trapezius
Lower trapezius
Serratus anterior
Activity before onset anterior deltoid
Activity after onset anterior deltoid
250
150
50
Onset
-50
-150
-250 ms
Anterior Deltoid
(B)
Infraspinatus
Upper trapezius
Lower trapezius
Serratus anterior
Activity before onset anterior deltoid
Activity after onset anterior deltoid
250
150
50
Onset
-50
-150
-250 ms
Anterior Deltoid
(C)
Infraspinatus
Upper trapezius
Lower trapezius
Serratus anterior
Activity before onset anterior deltoid
Activity after onset anterior deltoid
400
300
200
100
Onset
-100
-200
-300
-400 ms
Anterior Deltoid
: Stroke patients with shoulder pain; : Stroke patients without shoulder pain; : Controls

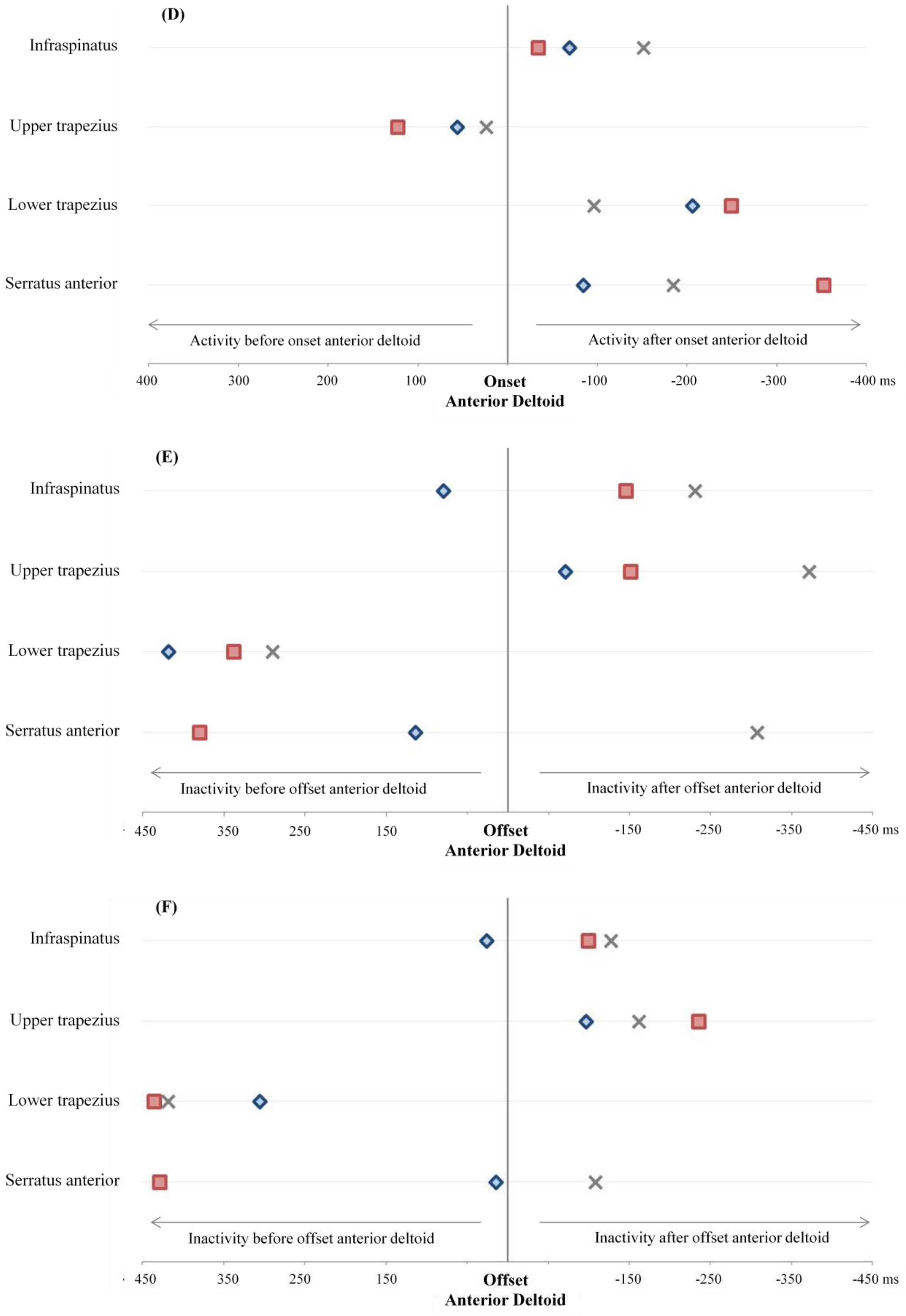

■ : Stroke patients with shoulder pain; ✕ : Stroke patients without shoulder pain; ◇: Controls

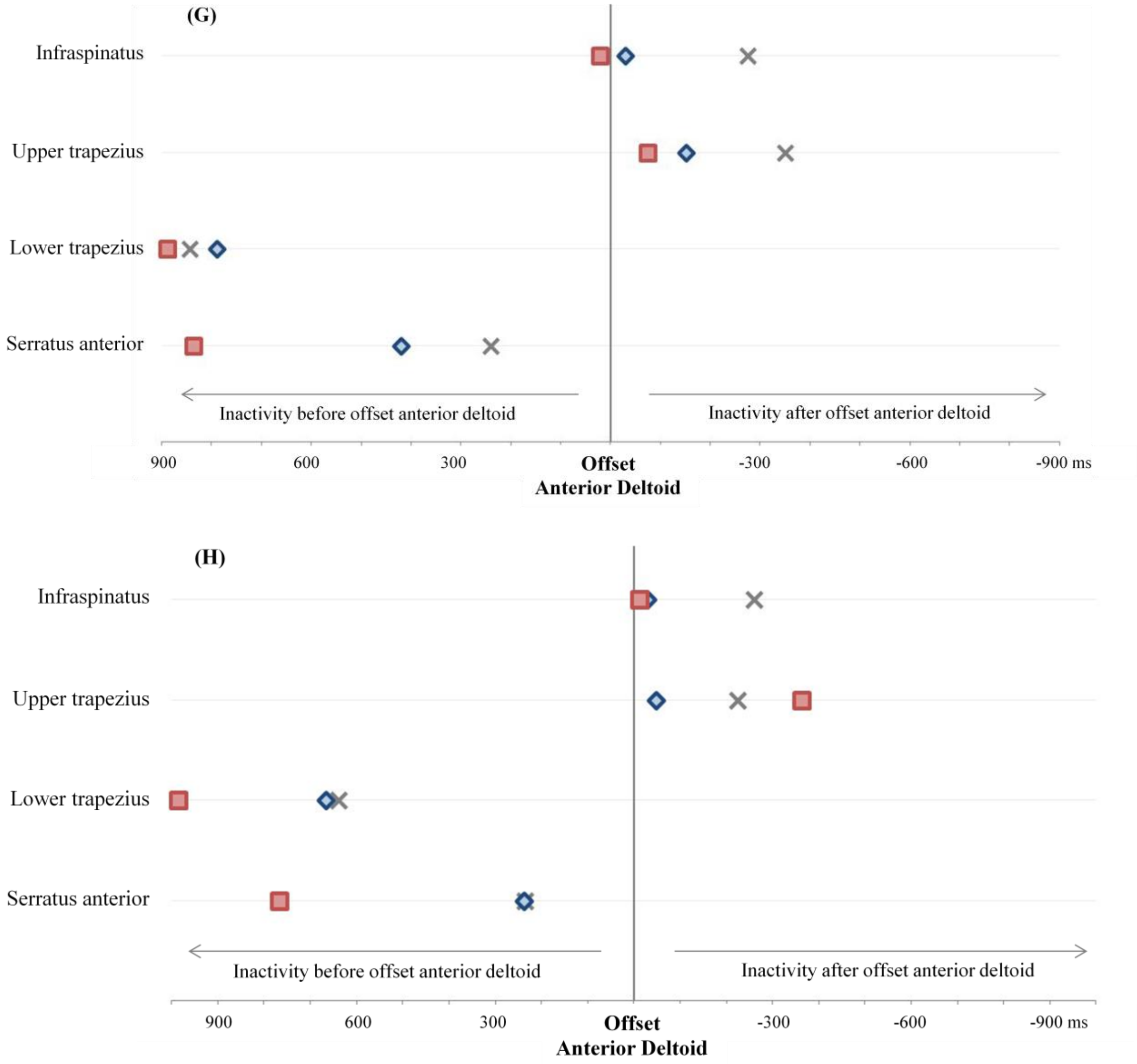

■: Stroke patients with shoulder pain; ✕ : Stroke patients without shoulder pain; ◆: Controls

Average onset (A-D) and offset (E-H) relative to anterior deltoid onset and offset in 45°, unloaded anteflexion (A, E); 45°, loaded anteflexion (B, F); full range, unloaded anteflexion (C, G); full range, loaded anteflexion (D, H)
